# Safety and efficacy of neoadjuvant chemoradiotherapy with moderately hypofractionated intensity‐modulated radiotherapy for resectable pancreatic cancer: A prospective, open‐label, phase II study

**DOI:** 10.1002/cam4.6470

**Published:** 2023-08-30

**Authors:** Toshihiko Masui, Kazuyuki Nagai, Takayuki Anazawa, Yosuke Kasai, Akitada Yogo, Michio Yoshimura, Takashi Mizowaki, Norimitsu Uza, Akihisa Fukuda, Shigemi Matsumoto, Masashi Kanai, Hiroyoshi Isoda, Yoshiya Kawaguchi, Shinji Uemoto, Etsuro Hatano

**Affiliations:** ^1^ Department of Surgery, Graduate School of Medicine Kyoto University Kyoto Japan; ^2^ Department of Radiation Oncology and Image‐Applied Therapy, Graduate School of Medicine Kyoto University Kyoto Japan; ^3^ Department of Gastroenterology and Hepatology Kyoto University Kyoto Japan; ^4^ Department of Real World Data Research and Development, Graduate School of Medicine Kyoto University Kyoto Japan; ^5^ Department of Clinical Oncology Kyoto University Kyoto Japan; ^6^ Department of Diagnostic Imaging and Nuclear Medicine Kyoto University Kyoto Japan

**Keywords:** chemoradiotherapy, IMRT, intensity‐modulated radiotherapy, neoadjuvant therapy, pancreatic cancer, surgery

## Abstract

**Background:**

Resectable pancreatic cancer (RPC) is potentially resectable on admission, and the impact of neoadjuvant therapy on these tumors is controversial. Moreover, the safety and efficacy of neoadjuvant chemoradiotherapy with moderately hypofractionated intensity‐modulated radiation therapy (NACIMRT) for RPC have not been studied. Here, we conducted a phase II study to evaluate the safety and efficacy of hypofractionated NACIMRT for RPC.

**Methods:**

A total of 54 RPC patients were enrolled and treated according to the study protocol. We used moderately hypofractionated (45 Gy in 15 fractions) IMRT with gemcitabine to shorten the duration of radiotherapy and reduce gastrointestinal toxicity. The primary endpoint was overall survival (OS), and we subsequently analyzed the microscopically margin‐negative resection (R0) rate, disease‐free survival (DFS), and histologic effects and safety of NACIMRT.

**Results:**

Median OS for the cohort was 40.0 months. Forty‐two patients (77.8%) underwent pancreatectomy after NACIMRT. Median DFS was 20.3 months. The R0 resection rate was 95.2% (40/42) per protocol and 85.2% (46/54) for the cohort. There were no intervention‐related deaths during the study period. Local treatment response, as assessed by the CAP classification, showed no residual tumor in 4.8% of patients. Overall, 23.9% of patients experienced CTCAE grade 3 or 4 during NACIMRT. Adjuvant therapy was initiated in 88% of patients undergoing resection. Postoperative complications grade ≥3b on the Clavien–Dindo scale occurred in 4.8% of patients. CA19‐9 level at enrollment was an independent prognostic factor for OS and DFS.

**Conclusions:**

This is the first prospective study of hypofractionated IMRT as neoadjuvant therapy for RPC. Hypofractionated NACIMRT for RPC could be safely introduced with a high induction rate of adjuvant chemotherapy, with an overall survival of 40.0 months.

## Graphical abstract

The median overall survival of resectable pancreatic cancer patients after neoadjuvant chemoradiotherpy with intensity‐modulated radiation therapy was 40.0 months without severe toxicity.

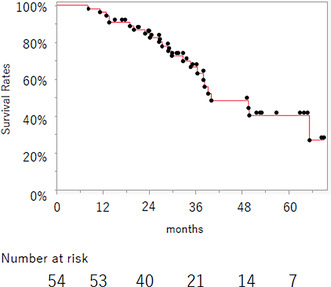

## 1 BACKGROUND

Pancreatic cancer has one of the poorest prognoses and highest mortality rates of any cancer.^1^ In Japan, pancreatic cancer is the fourth leading cause of cancer death, and its incidence is increasing. Although surgical resection results in a high cure rate, pancreatic cancer often recurs, and the 5‐year recurrence‐free survival rate in Japan is 15%.^2^

As defined by the National Comprehensive Cancer Network, pancreatic cancer is classified as resectable pancreatic cancer (RPC), borderline resectable pancreatic cancer (BRPC), and unresectable pancreatic cancer.^3^ RPC is considered if it meets the following criteria: (1) no arterial tumor contact with the celiac axis, superior mesenteric artery (SMA), or common hepatic artery (CHA); and (2) no tumor contact with the superior mesenteric vein (SMV) or portal vein or no more than 180° contact without venous contour irregularity. Neoadjuvant therapy for RPC is controversial because the lesions are resectable on admission. A recent randomized controlled study showed the benefits of neoadjuvant chemotherapy with gemcitabine plus S‐1^4^; however, another analysis showed a survival rate comparable to the non‐neoadjuvant approach in RPC in the PREOPANC trial.^5^ In addition, several studies are retrospective in nature and do not have information on how many RPC patients received neoadjuvant therapy,^6^ often resulting in an analysis of post‐resection survival rates rather than an intention‐to‐treat analysis.

Intensity‐modulated radiation therapy (IMRT) allows higher doses to be focused on an area with minimal exposure of normal tissue, thus maximizing the effect on the target area with minimal toxicity to surrounding organs. IMRT has been used for prostate and nasopharyngeal cancers, but precise irradiation is difficult because intra‐abdominal tissues move with respiration. In addition, there is little evidence to support the efficacy of IMRT for pancreatic cancer. Recently, moderately hypofractionated irradiation has been employed for breast^7^ and prostate cancers.^8^ The benefits of neoadjuvant hypofractionated irradiation include a shortened radiotherapy period, allowing patients to undergo surgery before adverse events (AEs) occur. We have reported that neoadjuvant with moderately hypofractionated IMRT results in favorable outcomes with minimal toxicity in BRPC patients^9^; however, the efficacy and safety of this technique in RPC patients have not been studied. Therefore, we prospectively conducted a phase II study to evaluate the safety and efficacy of neoadjuvant chemoradiotherapy with gemcitabine in combination with hypofractionated IMRT (NACIMRT) for RPC.

## 2 METHODS

### 2.1 Study design and patients

This study was conducted as a prospective phase II study of neoadjuvant chemotherapy with IMRT plus gemcitabine (jRCT1051190064) for RPC. The primary endpoint was overall survival (OS), and the secondary endpoints were the microscopically margin‐negative resection (R0) rate, disease‐free survival (DFS), and histologic effects and safety of NACIMRT. All patients diagnosed with RPC according to the NCCN 2016 guidelines^3^ at our hospital between June 2016 and August 2021 who provided informed consent were enrolled in this study. RPC status was assessed by multidetector‐row computed tomography (MDCT) using a multiphase contrast‐enhanced technique and evaluated by a multidisciplinary team consisting of physicians from the departments of surgery, gastroenterology, radiation oncology, clinical oncology, and diagnostic imaging. We retrospectively reviewed pre‐NACIMRT CT images of eligible patients and classified them according to the NCCN 2023 guidelines.^3^ All patients fell into the RPC category of the NCCN 2023 guidelines.

Inclusion criteria for this study were RPC patients with no arterial tumor contact with the CA, SMA, or CHA, and no tumor contact with the SMV or portal vein or contact of not more than 180° without venous contour irregularity on contrast‐enhanced MDCT. Other inclusion criteria were as follows: histologically confirmed pancreatic ductal adenocarcinoma; age between ≥20 and <80 years; ECOG performance status of 0 or 1; no distant metastases in the thorax, abdomen, or pelvis on dynamic contrast‐enhanced MDCT, positron emission tomography with 2‐deoxy‐2‐[^18^F]fluoro‐D‐glucose (FDG‐PET), and magnetic resonance imaging (MRI) with contrast medium of gadolinium‐ethoxybenzyl diethylenetriamine pentaacetic acid (EOB‐MRI); no pretreatment for current pancreatic cancer; and no hematologic or major organ dysfunction.

Exclusion criteria were as follows: interstitial pneumonitis; history of irradiation to the upper abdomen; severe comorbidities (heart failure, renal failure, liver failure, bleeding peptic ulcer, intestinal paralysis, intestinal obstruction, and uncontrolled diabetes); moderate or severe ascites or pleural effusion; history of active cancer (concurrent multiple cancers or heterogeneous multiple cancers with a disease‐free interval of <3 years); or expectant or nursing women. The Kyoto University Ethics Committee approved this study (YC1171), and each patient gave informed consent prior to participation.

### 2.2 Neoadjuvant chemoradiotherapy

**FIGURE 1:**
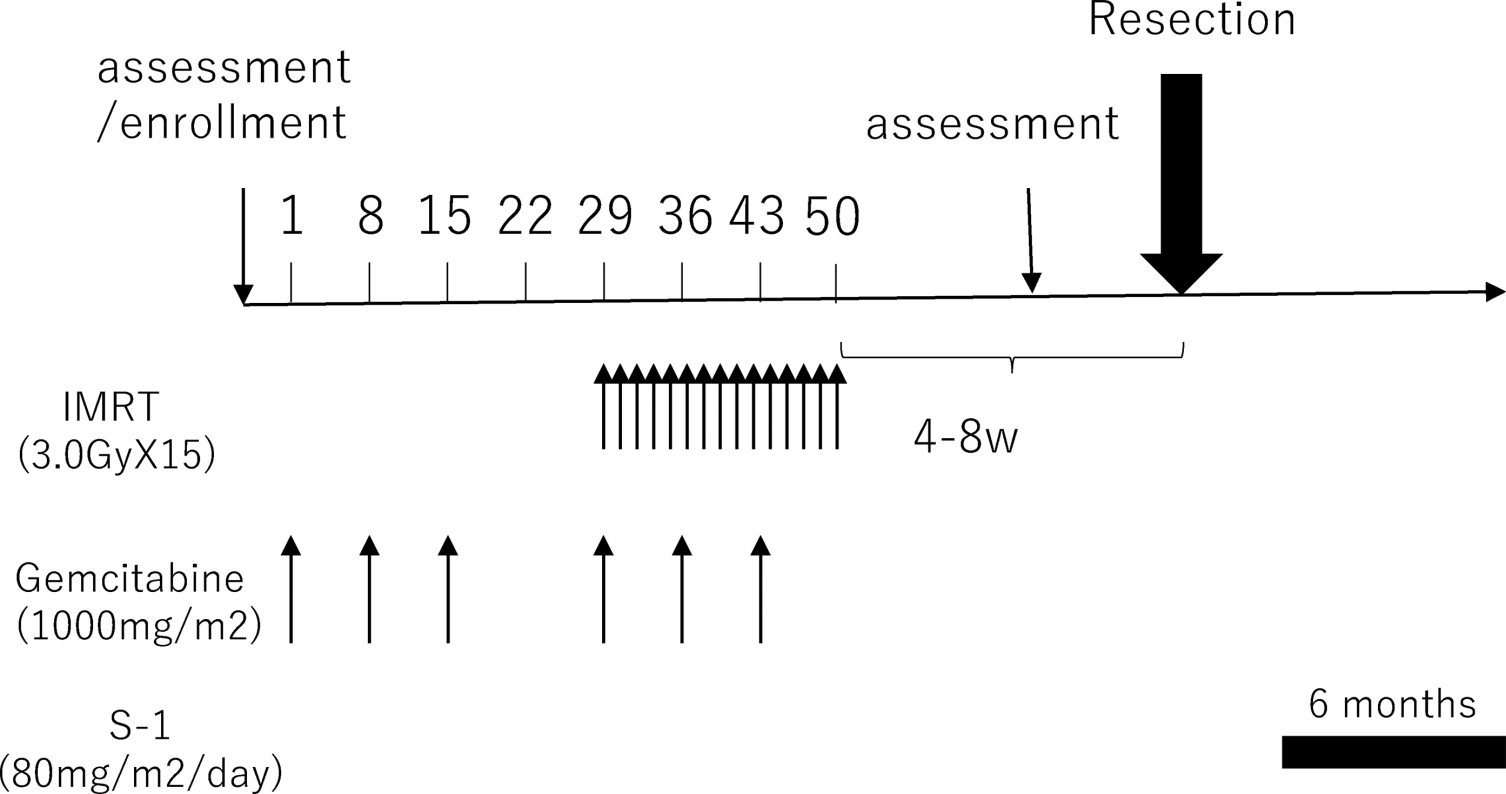
The treatment schedule consisted of induction chemotherapy with gemcitabine (1000 mg/m^2^), preoperative intensity‐modulated radiation therapy (IMRT) at 45 Gy (3.0 Gy/day, 5 times a week, 15 fractions), and concurrent intravenous gemcitabine (over 30 min on days 22, 29, and 36). Radiologic reassessment was performed 4–6 weeks after final irradiation.

The treatment flow of NACIMRT is shown in Figure 1. Following histologic confirmation of adenocarcinoma, patients were initially treated with gemcitabine at a dose of 1000 mg/m^2^ three times (Days 1, 8, and 22) before chemoradiotherapy. At the start of radiotherapy, gemcitabine was administered at a dose of 1000 mg/m^2^ (Days 1, 8, and 22) concurrently with IMRT. If grade 4 or worse neutropenia or thrombocytopenia occurred, chemotherapy was discontinued for 1 week. For IMRT planning, the gross tumor volume (GTV) included pancreatic tumors and lymph nodes >1 cm in diameter. The clinical target volume (CTV) included the retropancreatic regions between the CA and SMA in addition to the GTV plus a 5‐mm margin, according to our institutional contouring guidelines. The planning target volume (PTV) was defined as the CTV with a 5‐mm margin in all directions. GTV‐PRV and PTV‐PRV were volumes calculated by subtracting the stomach plus 10‐mm and the duodenum plus 5‐mm margins from the GTV and PTV, respectively. Organs at risk of injury were the stomach, duodenum, small intestine, colon, liver, kidneys, and spinal cord, which were delineated on expiratory‐phase CT. The prescribed dose was 45 Gy as D50% of GTV‐PRV and 42 Gy as D95% of PTV‐PRV (Dxx: The dose covering xx% of the target structure) in 15 fractions; PTV D98% ≥36 Gy was preferred.

Intensity‐modulated radiation therapy was performed with a treatment plan optimized for each patient. The breath‐hold method was used to minimize tumor motion, and cone‐beam CT was used before each treatment to determine daily setup errors. Radiation treatment was delivered using volumetric modulated arc therapy techniques.

### 2.3 Resection and adjuvant chemotherapy

Patients were evaluated for feasibility of resection within 4 weeks of neoadjuvant therapy using MDCT, EOB‐MRI, and FDG‐PET. In the absence of unresectable factors such as distant metastases, resection was attempted between 4 and 8 weeks after completion of neoadjuvant radiotherapy. Pancreaticoduodenectomy, distal or total pancreatectomy, and resection of all involved tissues were performed. Operative findings, surgical complications, and histopathology were recorded. S‐1 at a dose of 80 mg/m^2^/day was administered on Days 1–28 of a 42‐day cycle for 6 months as adjuvant chemotherapy, starting 4–8 weeks after resection.

### 2.4 Assessment

Resection margins were considered positive (R1) if malignant cells were observed on the surface of the resected specimen (0‐mm margin rule), in the plexus around the SMA or CHA, duodenum, bile duct, or retroperitoneal tissue. If a vein was concomitantly resected, the vein margin was also examined.

Follow‐up data up to December 2022 were analyzed. Patients were evaluated by contrast‐enhanced CT every 3 months for the first 2 years and every 6 months thereafter. A new low‐density mass in the peripancreatic and mesenteric root area was considered a locoregional recurrence. DFS was calculated as the time from the date of resection to recurrence and included off‐protocol patients who underwent resection (*n* = 7). OS was calculated as the time from enrollment to death. Radiological tumor response was evaluated by comparing tumor size on MDCT images before and after NACIMRT according to the Response Evaluation Criteria in Solid Tumors (RECIST v1.0), and pathological responses were evaluated by CAP grade. Toxicity events were recorded using the Common Terminology Criteria for Adverse Events (CTCAE) version 4.0.^10^ Weekly complete blood counts and liver function tests were performed from the start of radiotherapy until 2 weeks after the end of chemoradiotherapy. Pretreatment serum carbohydrate antigen 19‐9 (CA19‐9) levels were assessed after biliary drainage. No eligible patients were lost to follow‐up during the study period.

### 2.5 Statistics

The primary endpoint of OS and the secondary endpoints of DFS, response rate, pathologic response, R0 resection rate, surgical morbidity, and acute and late toxicity were evaluated 6 months after the completion of enrollment. The final dataset was carefully reviewed for clerical errors by three physicians (T.M., K.N., and T.A.). Data for continuous variables were expressed as median and range. Fisher's exact test and the Mann–Whitney *U* test were used for categorical and continuous data, respectively. Kaplan–Meier curves were created to estimate OS, and comparisons between groups were estimated using log‐rank tests. Multivariate Cox proportional hazards regression analysis was used to identify prognostic factors independently associated with survival. *p* < 0.05 was considered significant. In the regression analysis model for OS, multivariate analysis was performed using diameter, CEA, and CA19‐9, which have been reported as factors associated with OS. For DFS, multivariate analysis was performed using the forward selection method, and variables with *p* values less than 0.1 in univariate analysis were entered in addition to previously reported factors. All statistical analyses were performed on an intention‐to‐treat basis using JMP version 16.0 software (SAS Institute).

## 3 RESULTS

### 3.1 Patient characteristics

Fifty‐four patients were enrolled in this study. Baseline characteristics of the cohort are summarized in Table 1A. The median age was 70 years (range, 31–78), and 25 (46.3%) patients had tumors at the head of the pancreas. The median tumor size was 16.9 mm, and median carcinoembryonic antigen and CA19‐9 levels were within the normal range (2.75 [0.7–28.6] U/mL and 60.45 [0.6–4148.0] U/mL, respectively). The median neutrophil‐to‐lymphocyte ratio (NL ratio) before neoadjuvant treatment was 2.20 (1.0–4.5). In 18 patients with biliary obstruction, biliary drainage was performed by biliary stenting with a metallic stent.

**TABLE 1A:** Patient characteristics and post‐neoadjuvant chemoradiotherapy with moderately hypofractionated intensity‐modulated radiation therapy (NACIMRT) assessment.

Pre‐NACIMRT (at enrollment)	*N* = 54
Age (years)	70 (31–78)
Gender (male/female)	29/25
Head/body‐tail	25/29
Radiological tumor size (mm)	16.9 (7.0–44.0)
CEA before NACIMRT (U/mL)	2.75 (0.7–28.6)
CA19‐9 before NACIMRT (U/mL)	60.45 (0.6–4148.0)
NL ratio before NACIMRT	2.2 (1.0–4.5)
NAC incompletion due to adverse event	8/54 (14.8%)
Post‐NACIMRT (at reassement after NACIMRT)	*n* = 46 (85.2%)
Age (years)	70 (31–77)
Gender (male/female)	25/21
Relative dose intensity of gemcitabine	83.5% (66.6100.0)
Radiological tumor size after NACIMRT (mm)	13.0 (7.0–29.0)
CEA after NACIMRT (U/mL)	2.4 (0.7–25.5)
CEA ratio per pre‐NACIMRT	0.95 (0.06–4.05)
CA19‐9 after NACIMRT (U/mL)	23.6 (0.6–2121.0)
CA19‐9 ratio per pre‐NACIMRT	0.53 (0.02–2.51)
NL ratio after NACIMRT	3.1 (0.8–24.6)
RECIST ≥PR	13/46 (28.3%)
Grade 3/4 adverse event	10/46 (21.3%)

*Note*: Data are presented as median (range).

Abbreviations: NAC, neoadjuvant therapy; NL, neutrophil‐lymphocyte; RECIST, Response Evaluation Criteria in Solid Tumors.

### 3.2 Safety and clinical outcomes of neoadjuvant chemoradiotherapy with moderately hypofractionated intensity‐modulated radiation therapy

**FIGURE 2:**
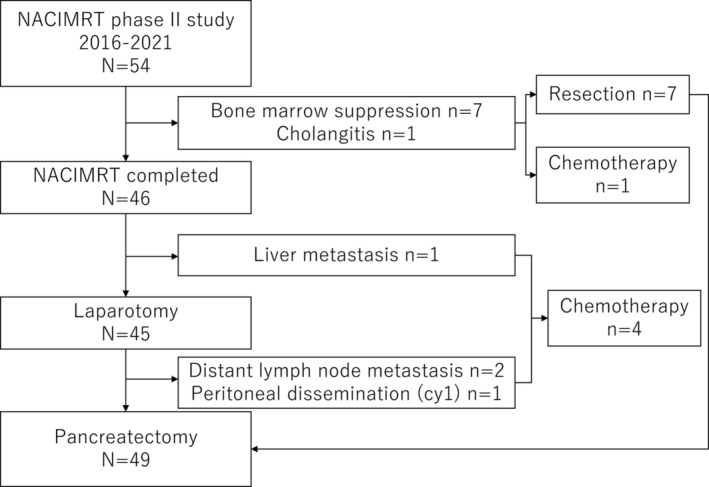
CONSORT diagram of the study flow. A total of 46 patients (85.2%) completed neoadjuvant chemoradiotherapy with moderately hypofractionated intensity‐modulated radiation therapy (NACIMRT) with gemcitabine, and 77.8% (42/54) of the patients underwent pancreatectomy after NACIMRT.

The study diagram is shown in Figure 2. The median time from staging MDCT to the start of neoadjuvant induction chemotherapy with gemcitabine was 14 days (range, 2–21). Of the 54 patients, 46 completed IMRT with gemcitabine (85.2%, 46/54). Seven patients discontinued due to severe bone marrow suppression and one patient due to cholangitis; seven patients underwent upfront surgery, and one patient was switched to gemcitabine with S‐1 due to liver metastases after neoadjuvant therapy. There were two patients with grade 3 leukopenia and six patients with grade 3 neutropenia; one patient with grade 3 leukopenia recovered after omitting concurrent gemcitabine, and the other seven patients did not recover within 2 weeks and underwent upfront resection. There were also one patient with grade 3 fatigue who received continuous infusion, one patient with grade 3 cholangitis who refused to continue NACIMRT and underwent chemotherapy, and one patient with grade 3 pancreatitis who developed during NACIMRT but recovered after the replacement of the stent in the pancreatic duct. The median relative dose intensity of gemcitabine was 83.5%. The incidence of grade 3/4 toxicity for NACIMRT was 16.7% (9/54; Table 1B). There were no cases of grade 3/4 gastroenterological toxicity.

**TABLE 1B:** Common Terminology Criteria for Adverse Events grade for neoadjuvant chemoradiotherapy with moderately hypofractionated intensity‐modulated radiation therapy comorbidities (*N* = 54).

Adverse event	Grade 1–2, *n* (%)	Grade 3, *n* (%)	Grade 4, *n* (%)
Anemia	2 (4)	0 (0)	0 (0)
Leukopenia	10 (19)	2 (4)	0 (0)
Neutropenia	8 (15)	6 (11)	0 (0)
Thrombocytopenia	5 (9)	0 (0)	0 (0)
Fatigue	9 (17)	1 (2)	0 (0)
Anorexia	13 (28)	0 (0)	0 (0)
Dyspepsia	1 (2)	0 (0)	0 (0)
Mucositis oral	2 (4)	0 (0)	0 (0)
Nausea	8 (15)	0 (0)	0 (0)
Abdominal pain	7 (13)	0 (0)	0 (0)
Constipation	3 (6)	0 (0)	0 (0)
Rash maculopapular	12 (22)	0 (0)	0 (0)
Cholangitis	0 (0)	1 (2)	0 (0)
Pancreatitis	0 (0)	1 (2)	0 (0)

In the group that completed NACIMRT, one patient developed liver metastasis, two patients showed distant lymph node metastasis, and one patient developed peritoneal dissemination; all were treated with chemotherapy. The median CA19‐9 level decreased from 60.45 (0.6–4148.0) U/mL to 23.6 (0.6–2121.0) U/mL, while the NL ratio increased from 2.2 (1.0–4.5) to 3.1 (0.8–24.6) after completion of NACIMRT. The objective radiologic response rate was 28.3%, and median radiographic tumor size decreased from 16.9 (7.0–44.0) mm to 13.0 (7.0–29.0) mm.

### 3.3 Surgical outcomes and pathological effects

The median interval from completion of IMRT to surgery was 26 days (range, 20–48 days). Of the 45 patients who underwent surgery, one had a positive washing cytology, and two had positive distant lymph node metastasis in the para‐aortic region on laparotomy, resulting in 42 patients (77.8%) undergoing pancreatectomy. In the 25 patients with pancreatic head cancer, 12 underwent biliary drainage with metallic stent, with no differences in operative time (521.7 min. vs. 517.8 min, *p* = 0.926), blood loss (577 mL vs. 691 mL, *p* = 0.565), or postoperative hospital stay (32.3 days vs. 23.4 days, *p* = 0.149) between patients with metal stent and those without. Clavien–Dindo 3a or higher was observed in one case in the metal stent group and in two cases in the non‐stent group, with no significant difference (*p* = 0.085).

R0 resection was achieved in 40/42 patients (95.2%; Table 2A). The overall R0 resection rate was 85.2% (46/54 patients including seven who did not complete NACIMRT and underwent pancreatectomy). According to the CAP classification, no residual tumor, <10% residual tumor, and >50% residual tumor were observed in 2 (4.7%), 7 (16.7%), and 23 (54.8%) patients, respectively. Pathologic lymph node metastasis was observed in 10 patients (23.8%).

**TABLE 2A:** Surgical outcome and pathological features.

Surgical outcome (per protocol)	*N* = 42
Type of procedure (PD/DP/TP)	19/23/0
Operation time (min)	450 (260–753)
Blood loss (mL)	425 (0–2610)
Laparoscopic surgery	13 (31.0%)
Clavien–Dindo ≥IIIa	6 (14.3%)
Pancreatic fistula (grade B or C)	7 (16.7%)
Delayed gastric emptying (grade B or C)	5 (11.9%)
Reoperation	1 (2.4%)
Duration of hospital stay	21 (8–48)
Inhospital death	0
Alb 1 month after resection (mg/dL)	3.4 (2.5–4.2)
ChE 1 month after resection (U/L)	229 (143–394)
Relative dose intensity of adjuvant S1 (%)	100 (0–100)
Introduction of S1 adjuvant therapy	37 (88.1%)
Completion of S1 adjuvant therapy	35 (83.3%)
Pathological features	
Tumor diameter (mm)	18.5 (0.0–48.0)
Positive LN metastasis	10 (23.8%)
CAP grade (3/2/1/0)	23/10/5/2
R0/R1	40/2 (95.2%)
Local recurrence/metastatic recurrence	2/21

*Note*: Data are presented as median (range).

Abbreviations: Alb, albumin; CAP, College of American Pathologists.; ChE, choline esterase; DP, distal pancreatectomy; LN, lymph node; PD, pancreaticoduodenectomy; S1, tegafur/gimeracil/oteracil; TP, total pancreatectomy.

Median albumin and cholinesterase levels were within normal ranges 1 month after surgery in the per‐protocol cohort. Of the 42 patients with R0/1 resection after completion of NACIMRT, 37 (88.1%) started postoperative adjuvant therapy with S‐1 within 6 weeks of resection (Table 2A); 35 (83.3%) completed 6 months of S‐1 treatment; and 26 (61.9%) received 100% of the S‐1 relative dose intensity. Five patients could not start adjuvant chemotherapy, two of whom had clinically relevant pancreatic fistulas; two refused to take S1; and one had delayed gastric emptying.

Postoperative Clavien–Dindo grade ≥3a AEs were observed in 14.3% of patients after NACIMRT, including five with clinically relevant postoperative pancreatic fistula; no inhospital deaths occurred (Table 2B). The median length of hospital stay after resection was 21.0 (8–48) days.

**TABLE 2B:** Clavien–Dindo classification for postoperative comorbidities (*n* = 42).

Clavien–Dindo classification	I, *n* (%)	II, *n* (%)	IIIa, *n* (%)	IIIb, *n* (%)	IVa, *n* (%)	IVb, *n* (%)	V, *n* (%)
Pancreatic fistula	7 (17)	2 (5)	4 (10)	1 (2)	0 (0)	0 (0)	0 (0)
Delayed gastric emptying	1 (2)	8 (19)	1 (2)	0 (0)	0 (0)	0 (0)	0 (0)
Wound infection	0 (0)	1 (2)	0 (0)	0 (0)	0 (0)	0 (0)	0 (0)
Intra‐abdominal abscess	0 (0)	1 (2)	0 (0)	0 (0)	0 (0)	0 (0)	0 (0)
Abdominal bleeding	0 (0)	0 (0)	0 (0)	1 (2)	0 (0)	0 (0)	0 (0)
Cholangitis	0 (0)	3 (7)	0 (0)	0 (0)	0 (0)	0 (0)	0 (0)
Chylous ascites	2 (5)	2 (5)	0 (0)	0 (0)	0 (0)	0 (0)	0 (0)
Diarrhea	0 (0)	2 (5)	0 (0)	0 (0)	0 (0)	0 (0)	0 (0)
Portal vein embolism	0 (0)	1 (2)	0 (0)	0 (0)	0 (0)	0 (0)	0 (0)
Edema	1 (2)	0 (0)	0 (0)	0 (0)	0 (0)	0 (0)	0 (0)
Pneumonia	0 (0)	1 (2)	0 (0)	0 (0)	0 (0)	0 (0)	0 (0)
Postoperative hospital stay, days (range)	21 (8–48)						

### 3.4 Survival

**FIGURE 3:**
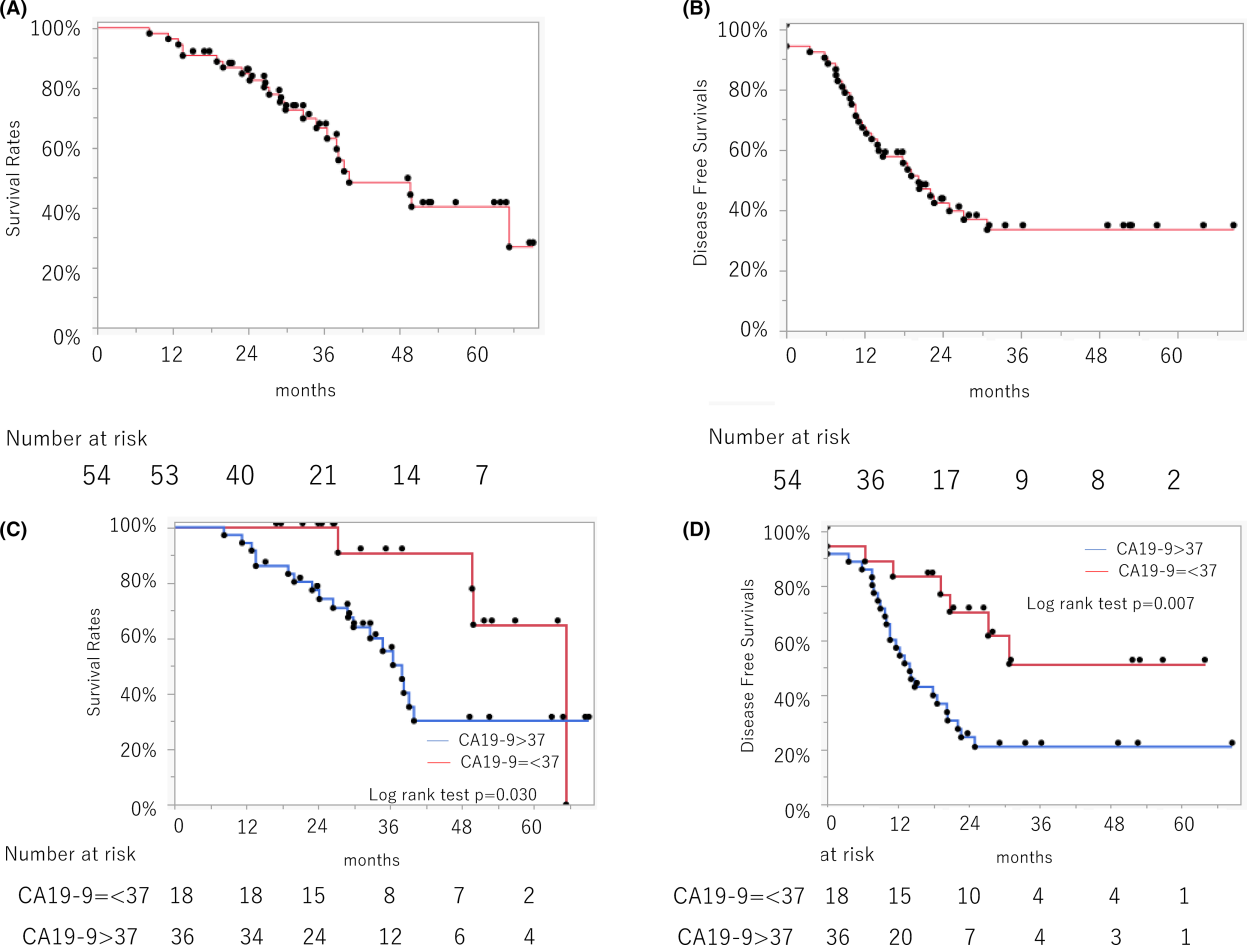
Intention‐to‐treat analyses of (A) overall survival and (B) disease‐free survival in 54 resectable pancreatic cancer patients. (C) Overall survival and (D) disease‐free survival according to CA19‐9 levels at enrollment. Patients with CA19‐9 level > 37 U/mL had significantly worse overall survival (*p* = 0.030) and disease‐free survival (*p* = 0.007) using the log‐rank test.

Kaplan–Meier plots for survival are shown in Figure 3. The median follow‐up for censored patients was 31.5 months. Median OS and DFS were 40.0 months (95% confidence interval [CI]: 36.5–n.a.; Figure 3A) and 20.3 months (95% CI: 13.1–30.8; Figure 3B), respectively. OS and DFS of patients with normal CA19‐9 levels were significantly longer than those with elevated CA19‐9 levels (OS: 65.3 vs. 38.0 months, *p* = 0.030; DFS: not reached vs. 14 months, *p* = 0.001; Figure 3C,D). To elucidate the independent factors related to prognosis, we analyzed factors affecting OS and identified normal serum CA19‐9 levels before NACIMRT to be independently associated with OS. For factors associated with DFS, univariate analysis showed that a normal initial CA19‐9 level, tumor diameter >20 mm, pathological N0 status, and R0 status were associated with DFS; however, only a normal initial CA19‐9 level and pathological N0 status were independent factors for DFS (Table 3).

**TABLE 3:** Factors associated with survivals.

	Univariate analysis			Multivariate analysis		
	Odds ratio	95% CI	*p* Value	Odds ratio	95% CI	*p* Value
Pre‐NACIMRT factors associated with overall survivals						
Age (>70 years)	0.99	0.95–1.04	0.581			
Male vs. female	0.78	0.34–1.80	0.57			
Ph vs. Pb or Pt	1.06	0.46–2.43	0.887			
CEA (>5.0 U/mL at enrollment)	1.57	0.66–3.75	0.31	1.30	0.54–3.15	0.560
CA19‐9 (>37 U/mL at enrollment)	8.07	2.80–23.31	0.001	3.55	1.13–11.17	0.030
Diameter (>20 mm at enrollment)	1.03	0.40–2.61	0.953	1.61	0.61–4.27	0.336
Factors associated with disease‐free survivals						
Operative time (>450 min)	1.10	0.48–2.50	0.820			
Blood loss (>425 mL)	0.86	0.38–1.95	0.720			
Clavien–Dindo >3a	1.87	0.44–8.02	0.398			
CA19‐9 (>37 U/mL at enrollment)	5.18	1.53–17.52	0.008	4.17	1.22–14.22	0.022
CA19‐9 (>37 U/mL before resection)	2.24	0.97–5.15	0.057	1.04	0.43–2.53	0.932
Diameter (>20 mm at enrollment)	1.70	1.04–2.73	0.029	1.00	0.41–2.43	0.996
Diameter (>20 mm at resection)	1.80	0.71–4.58	0.217			
CAP status ≤1	3.23	0.76–14.33	0.112			
Pathological LN positive	2.62	1.05–6.57	0.039	2.83	1.19–6.77	0.019
R0	3.90	1.16–13.07	0.027	2.11	0.40–11.20	0.380

Abbreviations: CAP, College of American Pathologists.; LN, lymph node; Pb, pancreas body; Ph, pancreas head; Pt, pancreas tail.

## 4 DISCUSSION

This study prospectively evaluated the safety and efficacy of neoadjuvant gemcitabine with moderately hypofractionated IMRT in 54 RPC patients who had undergone rigorous evaluation for resectability and distal metastases using EOB‐MRI, dynamic CT, and FDG‐PET. In our cohort, 46 patients completed IMRT with concurrent gemcitabine with no grade 4 or 5 AEs. In terms of efficacy, the median survival time (MST) of OS was 40.0 months, and R0 was achieved in 95.2% (40/42 patients) of the per‐protocol cohort and 85.2% (46/54 patients) of intention‐to‐treat analysis (ITT) including patients who did not complete NACIMRT. These results are better than those of our previous phase II study of gemcitabine plus IMRT as neoadjuvant therapy for BRPC‐A, in which 93.1% of the per‐protocol cohort achieved R0 resection and 55.3% of patients in ITT.^9^

In contrast to postoperative adjuvant therapy with various regimens for RPC, only a few prospective clinical studies have been conducted on neoadjuvant therapy for RPC due to the possibility of tumor resection on admission and concerns about disease progression after neoadjuvant therapy. Nevertheless, several neoadjuvant therapies have been conducted to date to improve survival in RPC patients. In these clinical studies, chemotherapy or chemoradiotherapy has been administrated. In the SWOG S1505 trial, in which FOLFIRINOX or gemcitabine plus nab‐paclitaxel was used as neoadjuvant therapy, 76% of initial patients underwent resection, and 85% of resected specimens were negative for R0 margins. Seventy‐eight percent of patients started and 63% completed adjuvant therapy.^11^ In another study, 182 RPC patients received gemcitabine plus S1 as neoadjuvant therapy, 86% of patients underwent resection, and a median OS of in ITT was 36.7 months.^4^ In the HOPS‐R01 trial using S‐1 as neoadjuvant therapy, 63% (31/49 patients) underwent pancreatectomy with a median OS of 35.5 months and ITT‐based progression‐free survival of 17.4 months.^12^ Regarding neoadjuvant chemoradiotherapy for RPC, Eguchi et al.^13^ applied 50.4 Gy of three‐dimensional (3D) conformal radiotherapy with gemcitabine plus S1 as neoadjuvant therapy to 62 RPC patients, 87% of patients were resected, and median OS was 55.3 months in ITT. In the PREOPANC study, a randomized phase III study of neoadjuvant gemcitabine plus radiotherapy for RPC and BRPC, hypofractionated 3D conformal radiotherapy (36 Gy in 15 fractions) with gemcitabine demonstrated a 66% resection rate with a median OS of 14.6 months in RPC patients.^5^

This study was conducted with the same purpose as the other studies of improving survival, with some differences in the approach to neoadjuvant therapy for RPC, and our approach has not been previously studied. We used IMRT to deliver therapeutic doses to affected tissue with minimal irradiation to surrounding normal organs compared to conventional radiotherapy. IMRT may have contributed to the low incidence of AEs in this study, resulting in a high completion rate of neoadjuvant chemoradiotherapy (85.2%, 46/54 patients). We used moderately hypofractionated schedules, although SBRT (5 fractions) or conventionally fractionated radiotherapy (25–30 fractions) has been used in neoadjuvant radiotherapy for pancreatic cancer.^14^ Hypofractionated irradiation may have contributed to the high completion rate in this study because it shortened the duration of irradiation, thereby reducing the incidence of AEs by chemotherapy and allowing patients to proceed to surgery more quickly. The incidence of gastroenterological AEs was low in this study, and the types of AEs appeared to be slightly different from other approaches. In the HOPS‐R01 trial using neoadjuvant S‐1,^12^ the proportion of patients with grade 3 gastroenterological toxicity (25%) was higher than that in this study (0%). In this study, 16% of patients experienced grade 1 to 2 abdominal pain, which could have been caused by IMRT. In the PREP‐02 phase III trial using neoadjuvant gemcitabine and S‐1,^4^ median OS was 39.2 months and no abdominal pain was observed, but grade 3 neutropenia developed in 35% of patients (11% in this study).

Besides the purpose of reducing toxicity, we used IMRT to increase the irradiation dose to the primary tumor. The biological effective dose at α/β of 10 (BED10) of 45 Gy in 15 fractions was 58.5 Gy, while the BED10 of the conventional standard treatment dose (45–50.4 Gy in 1.8‐Gy fractions) was 53.1–59.5 Gy. Given the shorter treatment duration but higher doses, the treatment intensity of hypofractionated therapy should be higher than that of standard treatment. We calculated the α/β of pancreatic cancer to be 10, which has not been established in the literature. If the α/β of pancreatic cancer were <10, the benefits of hypofractionated therapy would be even greater. Furthermore, the use of IMRT increased treatment intensity compared to the PREOPANC trial, which used conventional 3D conformal radiotherapy with 36 Gy in 15 fractions (BED10: 44.6 Gy). Moreover, our current strategy of increasing treatment intensity while minimizing AEs not only increases the R0 resection rate, but also increases the likelihood of maintaining local control even in inoperable cases, as shown by the favorable local control rate (1‐year locoregional progression‐free survival: 73.1%) in our previous study of hypofractionated IMRT for locally advanced pancreatic cancer.^15^

Adjuvant therapy with S‐1 is important to improve the long‐term survival of RPC patients. The JASPAC‐01 study with upfront surgery showed better survival (OS, 46.5 months in MST) with S‐1 compared to gemcitabine.^16^ In this study, postoperative adjuvant S‐1 therapy was administered to 88% of patients who underwent resection; 83.3% completed 6 months of adjuvant S‐1 therapy with the median relative dose intensity of 100%. Our current S‐1 induction/completion rates were higher, compared to those of other GS‐based neoadjuvant chemoradiation studies for RPC^13^ (70.3% and 55.5%, respectively).

In this study, elevated CA19‐9 levels (>37 U/mL) at enrollment were independently associated with OS and DFS. Recently, the term bio‐borderline RPC has been proposed to describe tumors with elevated CA19‐9 levels (>37 U/mL) at clinical stages I–II (TNM) that are anatomically resectable but have a poor prognosis.^17^, ^18^ The present data and this concept suggest that initial CA19‐9 is also important in RPC patients receiving neoadjuvant chemoradiotherapy. Furthermore, it suggests that the differences in initial CA19‐9 levels between cohorts may influence the outcome of clinical trials. Some studies argue that normalization of CA19‐9 levels after neoadjuvant therapy has been considered important for long‐term survival after resection.^19^ In our cohort, initial high CA19‐9 levels were more significantly associated with survivals rather than normalized CA19‐9. Because high CA19‐9 levels at enrollment were associated with those after neoadjuvant therapy (data not shown), our current neoadjuvant chemoradiotherapy may not be intense enough to normalize CA19‐9 levels regardless of the initial level. This is consistent with our previous study for BRPC with arterial abutment^9^ with the same neoadjuvant NACIMRT treatment, in which a serum CA19‐9 level ≥400 IU/mL was an independent prognostic factor but normalized CA19‐9 was not.

This study has several limitations. First, this was a single‐center study; standardizing IMRT treatment in a multicenter study is difficult because GTV and CTV must be designed as standard procedures according to tumor location, size, and vascular anatomy. Second, the current treatment protocol uses gemcitabine monotherapy as concurrent therapy, which is outdated for the newer doublet or triplet combination therapy. Given the recurrence rate of distant metastases (21/23, Table 2A), more potent chemotherapy, such as gemcitabine/nab‐paclitaxel, in combination with less toxic IMRT may be more effective. Third, because this is not a randomized clinical trial, it is difficult to compare results with other therapies. Future well‐designed randomized controlled trials should be prepared.

## 5 CONCLUSIONS

This is the first report of neoadjuvant with moderately hypofractionated IMRT for RPC, resulting in a median OS of 40.0 months and a median DFS of 20.3 months. RPC patients who had received neoadjuvant hypofractionated IMRT had high induction and completion rates of postoperative adjuvant chemotherapy, which may have an impact on post‐resection survival.

## AUTHOR CONTRIBUTIONS

**Toshihiko Masui:** Conceptualization (lead); data curation (equal); formal analysis (lead); funding acquisition (lead); methodology (equal); writing – original draft (lead). **Kazuyuki Nagai:** Data curation (equal); formal analysis (equal). **Takayuki Anazawa:** Data curation (equal); formal analysis (equal). **Yosuke Kasai:** Investigation (supporting); validation (supporting). **Akitada Yogo:** Data curation (supporting); methodology (supporting). **Michio Yoshimura:** Conceptualization (supporting); methodology (equal); writing – review and editing (supporting). **Takashi Mizowaki:** Conceptualization (supporting); methodology (supporting). **Norimitsu Uza:** Data curation (supporting); validation (supporting); writing – review and editing (supporting). **Akihisa Fukuda:** Data curation (supporting); validation (supporting); writing – review and editing (supporting). **Shigemi Matsumoto:** Conceptualization (supporting); writing – review and editing (supporting). **Masashi Kanai:** Conceptualization (supporting); writing – review and editing (supporting). **Hiroyoshi Isoda:** Conceptualization (supporting); methodology (supporting). **Yoshiya Kawaguchi:** Conceptualization (equal); supervision (supporting). **Shinji Uemoto:** Conceptualization (supporting); supervision (equal). **Etsuro Hatano:** Conceptualization (supporting); resources (supporting); supervision (equal).

## FUNDING INFORMATION

This work was supported by JSPS KAKENHI Grant Number 21K08732.

## CONFLICT OF INTEREST STATEMENT

The authors have no competing interests to declare regarding this study.

## ETHICS STATEMENT

The study protocol has been approved by the Ethics Committee of Kyoto University Graduate School and Faculty of Medicine (YC1171) along with all necessary institutional and jurisdictional ethics committees. This study was performed in accordance with the ethical standards of the Declaration of Helsinki and its later amendments. Written informed consent for participation in this study was obtained from each participant.

## PATIENT CONSENT STATEMENT

No identifiable data are presented.

## TRIAL REGISTRATION

Japan Registry of Clinical Trials (jRCT): https://jrct.niph.go.jp/en‐latest‐detail/jRCT1051190064. Registration number: jRCT1051190064. Date of first registration: 06/01/2016.

## Data Availability

The datasets used and/or analyzed during the current study are available from the corresponding author on reasonable request.
